# Venetoclax and Decitabine for T/Myeloid Mixed-Phenotype Acute Leukemia Not Otherwise Specified (MPAL NOS)

**DOI:** 10.1155/2020/8811673

**Published:** 2020-10-10

**Authors:** Heather Klocke, Zhao Ming Dong, Craig O'Brien, Nicholas Burwick, Robert E. Richard, Daniel Y. Wu, Thomas R. Chauncey, Solomon A. Graf

**Affiliations:** ^1^Veterans Affairs Puget Sound Health Care System, Seattle, WA, USA; ^2^Department of Pathology, University of Washington Medicine, Seattle, WA, USA; ^3^Division of Hematology, University of Washington Medicine, Seattle, WA, USA; ^4^Division of Medical Oncology, University of Washington Medicine, Seattle, WA, USA; ^5^Clinical Research Division, Fred Hutch Cancer Research Center, Seattle, WA, USA

## Abstract

T/myeloid mixed-phenotype acute leukemia not otherwise specified (MPAL NOS) is an uncommon and aggressive leukemia without well-established treatment guidelines, particularly when relapsed. Venetoclax plus a hypomethylating agent offers a promising option in this situation since studies support its use in both acute myeloid and, albeit with fewer data to date, acute T-cell-lymphoblastic leukemias. We report the successful eradication of T/myeloid MPAL NOS relapsed after allogeneic stem cell transplant with venetoclax plus decitabine. A consolidative allogeneic stem cell transplant from a second donor was subsequently performed, and the patient remained without evidence of disease more than one year later. Further investigation is indicated to evaluate venetoclax combined with hypomethylating agents and/or other therapies for the management of T/myeloid MPAL NOS.

## 1. Introduction

Mixed-phenotype acute leukemia (MPAL) is characterized by leukemic blasts expressing antigens of more than one lineage to such a degree that it is not possible to assign one lineage with certainty. MPAL and acute undifferentiated leukemia (which shows no lineage-specific antigen) constitute a group of acute leukemias of ambiguous lineage. MPAL are uncommon, accounting for <5% of acute leukemias [1, 2]. MPAL T/myeloid NOS (T/myeloid MPAL) fulfills criteria for both T-cell and myeloid lineage without specific genetic abnormalities such as BCR-ABL1 or KMT2A rearrangement and is one of 5 MPAL subtypes recognized by 2016 World Health Organization classification [3]. T/myeloid MPAL is considered a high-risk acute leukemia, and limited data guide its management at diagnosis and relapse [4]. Venetoclax plus a hypomethylating agent (HMA) was approved for use by the Food and Drug Administration in November 2018 for older or relatively frail patients with newly diagnosed acute myeloid leukemia (AML) [5]. We report here the first, to our knowledge, successful application of venetoclax plus HMA for relapsed T/myeloid MPAL.

## 2. Case Report

**Figure 1:**
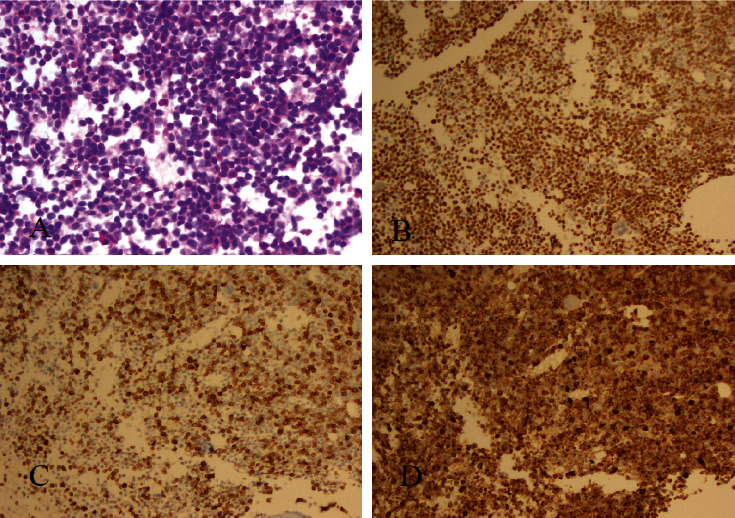
Bone marrow biopsy with T/myeloid mixed-phenotype acute leukemia as demonstrated by H&E stain with 80% blasts. (a) 200x and immunohistochemical stains for terminal deoxynucleotidyl transferase. (b) DAB, 200x, cytoplasmic CD3 with anti-CD3 (2GV6) rabbit monoclonal antibody against the epsilon chain. (c) DAB, 200x, and myeloperoxidase. (d) DAB, 200x.

A previously healthy 65-year-old man presented to an outside hospital with fatigue, folliculitis, easy bruising, vision changes, and decreased hearing. He had a normal coagulation screen; however, he was found to have cytopenias with a white blood cell count of 5.5 K/*µ*L that included approximately 50% blast forms, hemoglobin of 10.0 g/dL, and platelet count of 78 K/*µ*L. Marrow showed 70–80% blasts positive for CD34, terminal deoxynucleotidyl transferase (TdT), CD3, myeloperoxidase (MPO), and CD5, and negative for PAX-5 and c-Kit (Figure 1). Flow cytometry (FC) demonstrated the blasts to express markers specific for myeloid (cytoplasmic MPO) and T-lymphoid (cytoplasmic CD3) lineages. Blasts were also positive for CD5, CD7, CD10, CD34, CD11b, CD33, and TdT on FC. Cytogenetics were normal, and no abnormalities were identified on 200 interphase cells examined by fluorescence in-situ hybridization: specifically, analysis showed no evidence of 3q21.3q26.2 translocation or inversion, deletion 5q31, monosomy 7, deletion 7q31, RUNX1T1-RUNX1 translocation, KMT2A rearrangement, CBFB rearrangement, or PML/RARA translocation. Next generation sequencing identified the following mutations: DNMT3A c.2206C > T (variant allele frequency (VAF) 43.9%); DNMT3A c.1755dup (VAF 36.5%); IDH1 c.394C > T (VAF 44.0%); CBL c.1227 + 2T > C (VAF 51.6%); and NOTCH 1 c.5023_5025del (VAF 13.9%). Together, findings established the diagnosis of T/myeloid MPAL. Cerebrospinal fluid was negative by cytology and FC.

Induction treatment was given with 3 half cycles (1A, 1B, and 2A) of cyclophosphamide, vincristine, doxorubicin, and dexamethasone alternating with methotrexate and cytarabine (hyper-CVAD) and intrathecal (IT) prophylaxis [6]. This achieved a complete remission (CR), with bone marrow biopsy after cycle 1A, showing no evidence of disease by morphology or FC and no circulating blasts in the peripheral blood. He was transferred to our facility for consideration of consolidation stem cell transplantation. A repeat bone marrow exam was performed and demonstrated continued remission. The patient proceeded to a matched related donor allogeneic stem cell transplantation (allo-SCT) from his brother with reduced intensity conditioning (RIC) (fludarabine 30 mg/m^2^ daily for 3 consecutive days (Flu) and 200 cGy total body irradiation (TBI)) with tacrolimus and mycophenolate mofetil graft versus host disease (GvHD) prophylaxis [7]. Marrow on day 28 posttransplant showed morphologic remission with 0.06% blasts by FC. Markers used to detect MRD by FC included CD45, CD34, CD38, CD48, CD56, CD71, CD117, CD123, CD5, CD7, CD33, cCD3, sCD3, CD4, CD8, CD13, CD14, CD15, CD16, CD19, CD64, and HLA-DR. A repeat marrow one week later showed an increase in blasts to 0.1% by FC, and chimerism analysis showed donor CD3 of 40%, CD33 of 85%, and CD56 of 71%. His immunosuppression was tapered and completely discontinued on day 47, and he received donor-lymphocyte infusion with pentostatin on day 50 posttransplant. At day 18 post DLI blasts had increased to 6% by FC; by morphology, a population of 5% TDT + CD34+ blasts were shown. The patient was next treated with one cycle of intensive reinduction using cladribine, cytarabine, filgrastim, and mitoxantrone (CLAG-M) [8] plus vincristine and dexamethasone. This was complicated by neutropenic sepsis requiring prolonged intensive care, but the patient made a full recovery, and restaging bone marrow biopsy showed a CR by morphology and FC. A second allogeneic stem cell transplant was planned, but shortly beforehand, the patient developed grade 2 GvHD of the skin and was treated successfully with oral corticosteroids. Given that he was still in CR by FC and hoping the GvHD would correspond to increased graft versus leukemia, the 2^nd^ transplant was postponed. Unfortunately, repeat bone marrow exam approximately 3 months later confirmed low-level relapse detectable by FC. Reinduction with 2 cycles of CLAG-M plus vincristine and dexamethasone (with mitoxantrone omitted from the 2^nd^ cycle) was again administered but associated with substantially increased toxicity to the patient's functional status and a prolonged length of recovery. CR by morphology and FC was briefly achieved before disease was identified at a level of 0.9% by FC on day 441 posttransplant.

At this juncture, the patient was >1-year post-allo-SCT with relapsed T/myeloid MPAL despite several rounds of high-intensity chemotherapy. He was reasonably fit, evidenced by his regular exercise bicycling regimen, but had shown declining tolerance of successive cytotoxic chemotherapy regimens. He had no clinically active GvHD. Given the paucity of published data to inform this clinical scenario, we presented 3 options for management that included (1) directly pursuing a 2^nd^ allo-SCT using a different donor; (2) additional conventional multiagent chemotherapy; or (3) venetoclax plus HMA. He began venetoclax 400 mg by mouth daily and decitabine 20 mg/m^2^ intravenous daily on days 1–5 in 28 day cycles. No dose ramp-up of venetoclax was given in the setting of low disease burden. Because of grade 4 neutropenia and grade 4 thrombocytopenia, venetoclax was stopped on day 25. Both cell lines recovered by day 35, and a bone marrow exam at that time showed CR by morphology and FC. A 2^nd^ cycle was given, with venetoclax dose-reduced to 100 mg daily. A full 28 days of venetoclax was administered with recovery of neutrophils and platelets on day 37. Other than cytopenias, the regimen was well tolerated. He proceeded to a 2^nd^ SCT from a matched unrelated donor allograft with Flu/TBI RIC and cyclosporine, mycophenolate mofetil, and sirolimus GvHD prophylaxis [9]. Engraftment of neutrophils occurred on day 19 after transplantation, and no grade >1 GvHD has been observed to date. At his most recent follow-up 1 year after his 2^nd^ allo-SCT he remains in CR by morphology and FC with CD3, CD33, and CD56 compartments entirely of donor origin.

## 3. Discussion

Relapsed T/myeloid MPAL, including after allo-SCT, carries a poor prognosis with no established treatment protocols. Generally, strategies are extrapolated from studies of more common acute leukemias [4]. Consolidative second allo-SCT can be beneficial to select patients with relapsed AML [10]. In our case, a 2^nd^ allo-SCT was considered a reasonable approach if CR without detectable disease could be achieved since the patient had a good functional status, few comorbidities, and a different matched donor. Venetoclax, an inhibitor of the antiapoptotic protein BCL-2, is a generally well-tolerated oncolytic with synergistic antileukemic activity when combined with an HMA [5]. Though limited prospective data detail the regimen's use in the relapsed AML setting, it appears to have significant activity, with CR rates as high as 40% in a retrospective analysis of 219 patients with relapsed AML, myelodysplastic syndrome, or blastic plasmacytoid dendritic cell neoplasm [11]. Still fewer data exist on the use of venetoclax plus HMA in relapsed T-cell leukemias [12]. A series of 12 patients with relapsed T-cell leukemia treated with venetoclax plus various chemotherapies (included an HMA in 3) was recently published and showed an impressive response rate of 60% [13]. In addition, emerging data from recent and ongoing clinical trials have shown promising outcomes with venetoclax and an HMA in cases of acute myeloid leukemia with an IDH1/2 mutation; this may have particular relevance to our case in which a mutation in IDH1 was detected at diagnosis [14].

## 4. Conclusion

To our knowledge, this is the first report of venetoclax plus HMA for relapsed T/myeloid MPAL. The regimen was a successful bridge to 2^nd^ allo-SCT  and well-tolerated aside from prolonged cytopenias in the setting of repeated prior cytotoxic chemotherapy regimens. Regimens for MPAL are typically designed to treat both myeloid and lymphoid lineages; therefore, based on publications of retrospective series to date, venetoclax plus HMA offers a promising novel approach to this clinically challenging entity [11, 13]. Future research is required to further explore this strategy and combine venetoclax with other agents active in T/myeloid MPAL.
